# Supplementary material for: Soil microbial responses to multiple global change factors as assessed by metagenomics
Source: Nat Commun. 2025 May 31;16:5058. doi: 10.1038/s41467-025-60390-4 (PMC12125317; doi:10.1038/s41467-025-60390-4)
Supplement: Supplementary file 1 — Supplementary Information [file 41467_2025_60390_MOESM1_ESM.pdf]

Supplementary Information

Soil microbial responses to multiple global change factors as assessed by metagenomics

Álvaro Rodríguez del Río[1,*], Stefan Scheu[2,3] and Matthias C. Rillig[1,4]

[1] *Institute of Biology, Freie Universität Berlin, 14195, Berlin, Germany*
[2] *JFB Institute of Zoology and Anthropology, University of Göttingen, 37073 Göttingen, Germany*
[3] *Centre of Biodiversity and Sustainable Land Use, University of Göttingen, 37077 Göttingen, Germany*
[4] *Berlin-Brandenburg Institute of Advanced Biodiversity Research (BBIB), 14195, Berlin, Germany*
[*] *Corresponding author. Email: alvarordr94@gmail.com*

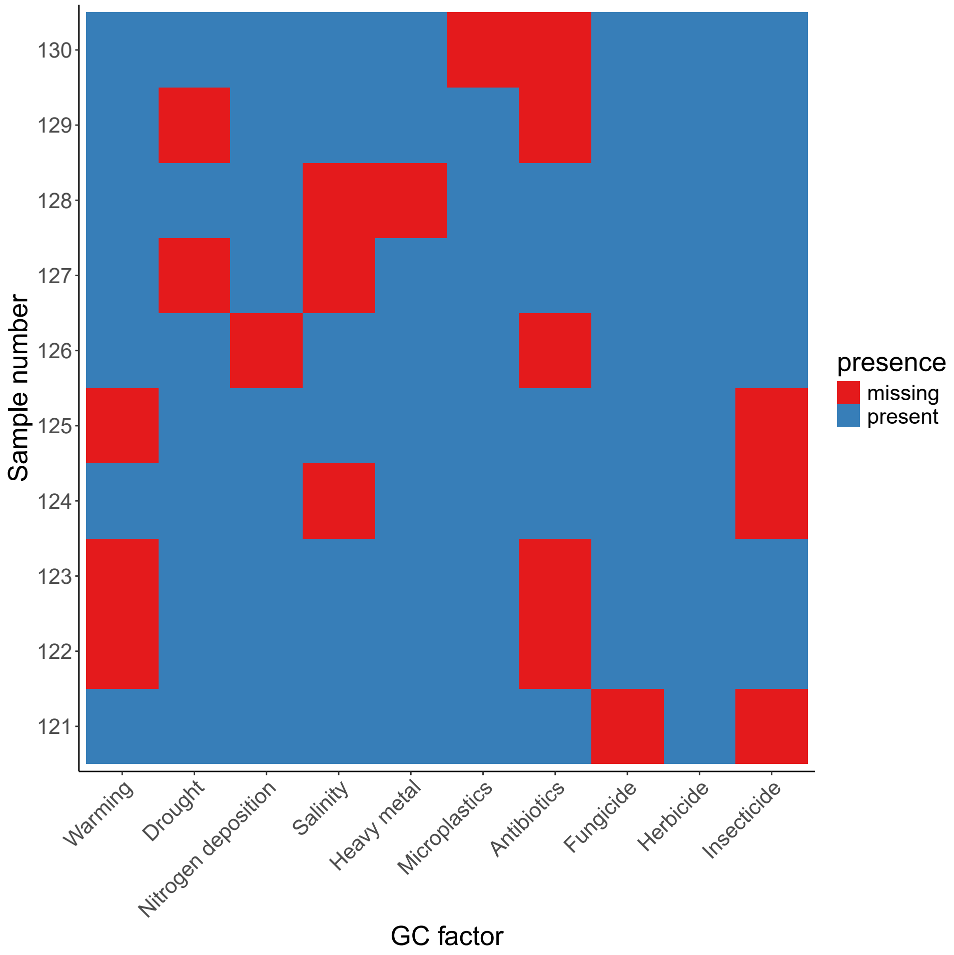

Supplementary Fig. 1. Random factor combinations included in each of the 8 GC factor samples.

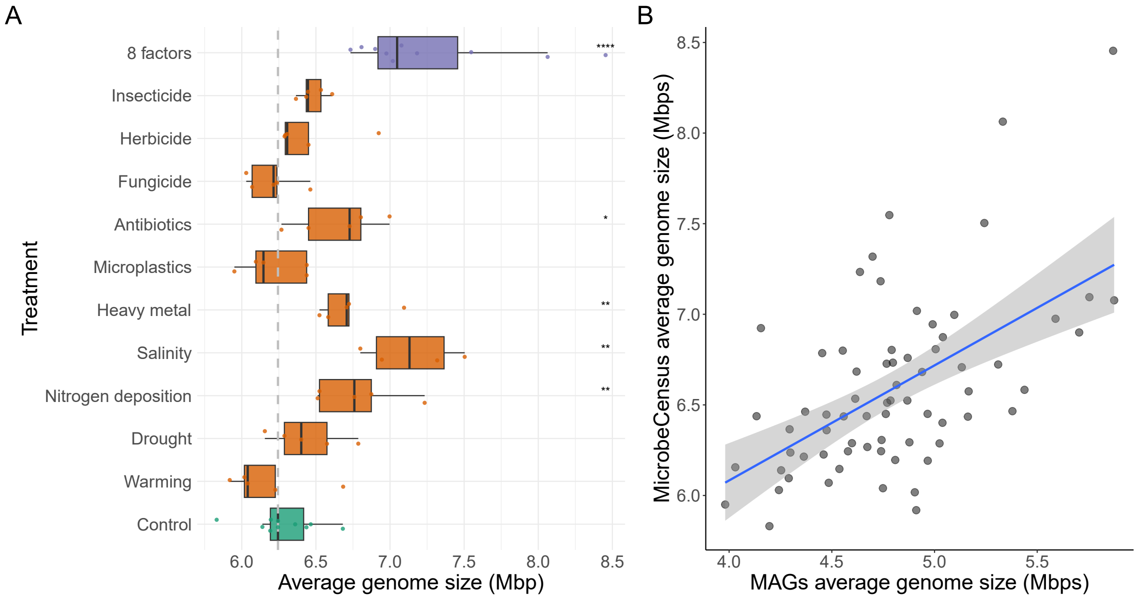

Supplementary Fig. 2. Genome size differences across samples. A) Average genome size calculated by MicrobeCensus[1]. Data are represented as boxplots in which the middle line is the median, the lower and upper hinges correspond to the first and third quartiles, the upper whisker extends from the hinge to the highest value no further than 1.5 × interquartile range (IQR) from the hinge and the lower whisker extends from the hinge to the lowest value no further than 1.5 × IQR of the hinge. Asterisks represent different significance levels obtained after a Two-sided Wilcoxon test comparing control samples with the samples to which GC treatments were applied; * indicate $p <= 0.05$, ** $p <= 0.01$, *** $p <= 0.001$ and **** $p <= 0.0001$. 10 Control samples, 5 samples for each individual GC treatment, and 10 8-factor samples were considered in the statistical analyses. B) Correlation between average genome size of the MAGs computed on each sample, and the MicrobiomeCensus average genome size. Blue lines represent linear regression lines, and shaded areas indicate 95% confidence intervals.

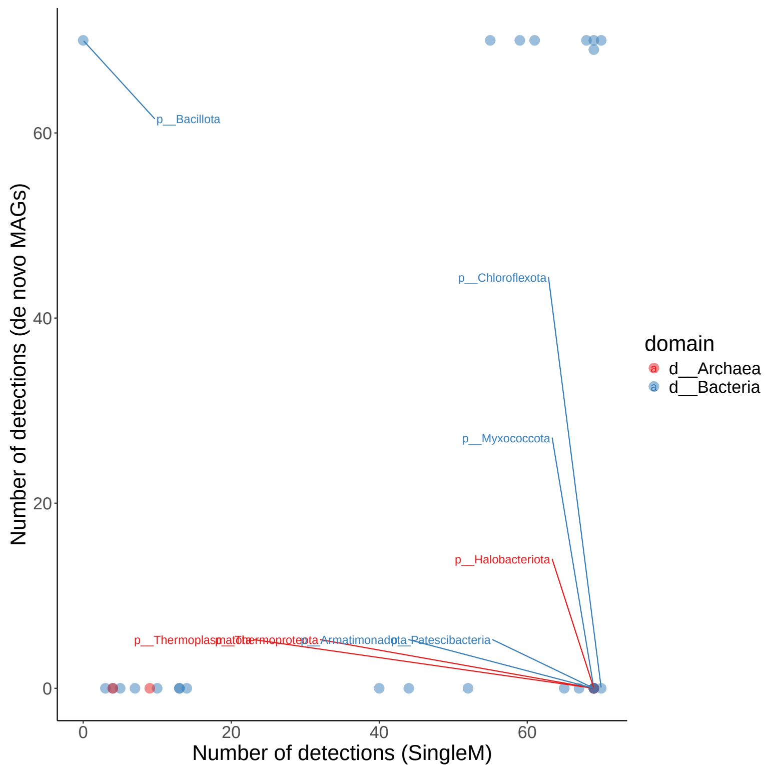

Supplementary Fig. 3. Comparison between the phyla detected by SingleM [2] (x-axis) and the phyla included in our MAG collection (y-axis).

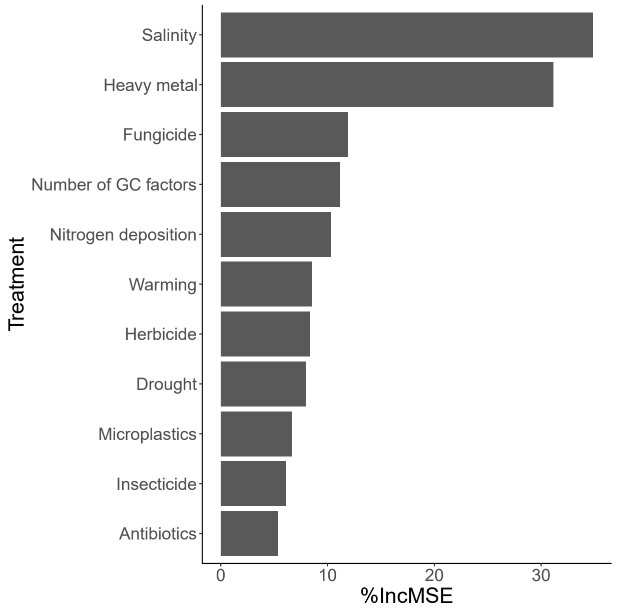

Supplementary Fig. 4. Mean decrease accuracy (%IncMSE) in bacterial diversity prediction by a random forest regression model of each GC factor, and the number of GC factors.

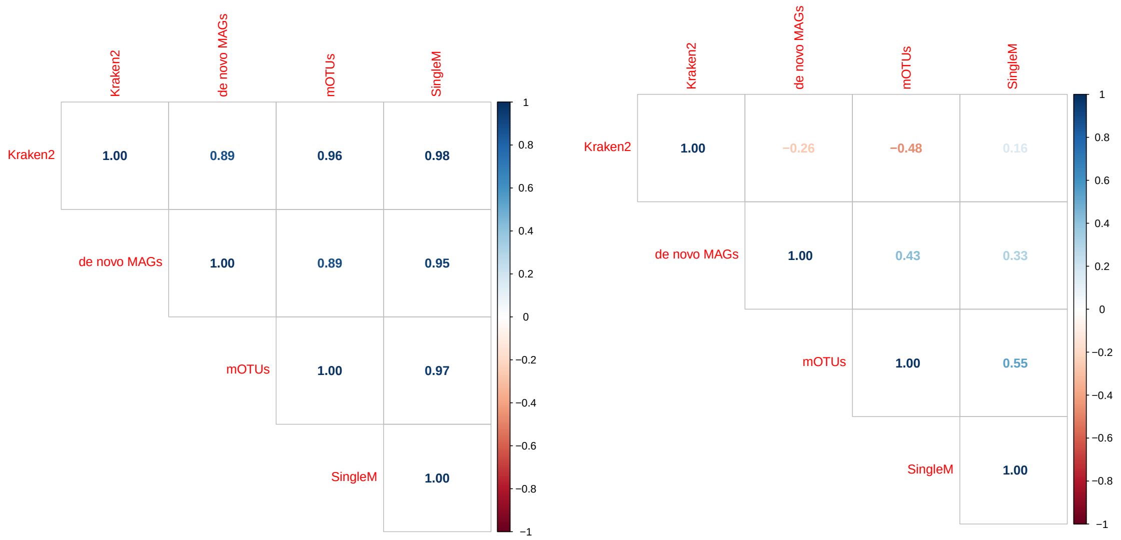

Supplementary Fig. 5. Correlation across bacterial composition (left) and diversity (right) measured by different taxonomic profiling methods. Kraken2 [3] (read taxonomic classification) provides different alpha diversity patterns than the rest of methods.

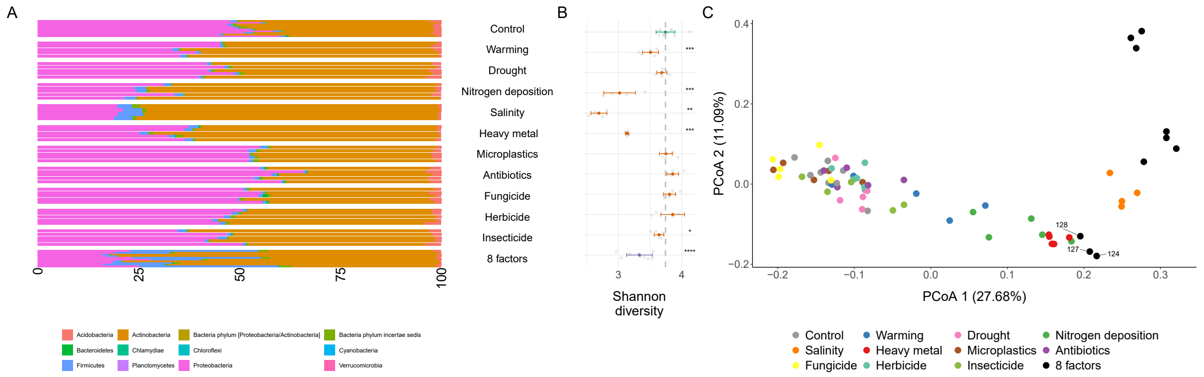

Supplementary Fig. 6. Community composition analysis taking mOTUs [4] taxonomic profiles as a reference. A) Taxonomic profile collapsed to the phylum level. B) Shannon diversity index, per treatment. Thick points represent the median values, and bars indicate standard deviation intervals. Asterisks represent different significance levels obtained after a Two-sided Wilcoxon test comparing control samples with the samples to which GC treatments were applied; * indicate p <= 0.05, ** p <= 0.01, *** p <= 0.001 and **** p <= 0.0001. 10 Control samples, 5 samples for each individual GC treatment, and 10 8-factor samples were considered in the statistical analyses. C) Principal coordinate analysis based on the taxonomic annotations of the reference viral MAGs. We indicate 8 factor samples not including the salinity treatment (124, 127, 128).

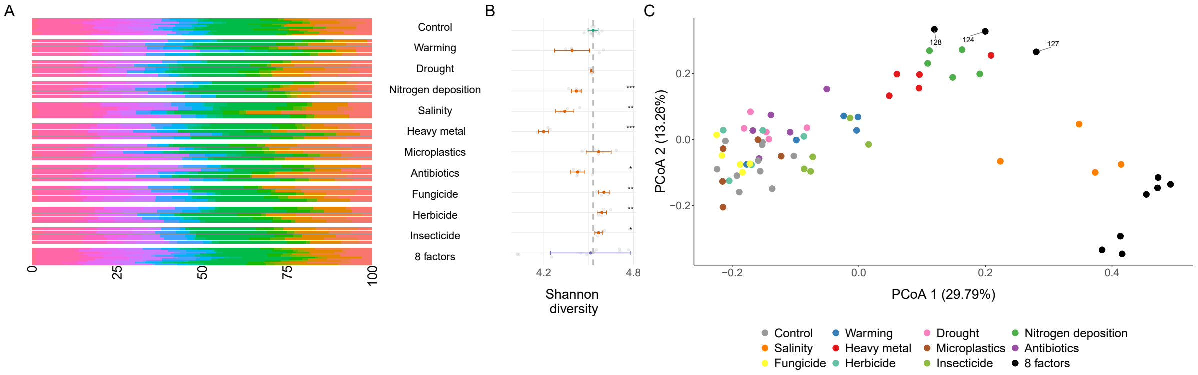

Supplementary Fig. 7. Community composition analysis taking SingleM [2] taxonomic profiles as a reference. A) Taxonomic profile collapsed to the phylum level. B) Shannon diversity index, per treatment. Thick points represent the median values, and bars indicate standard deviations. Asterisks represent different significance levels obtained after a Two-sided Wilcoxon test comparing control samples with the samples to which GC treatments were applied; * indicate p <= 0.05, ** p <= 0.01, *** p <= 0.001 and **** p <= 0.0001. 10 Control samples, 5 samples for each individual GC treatment, and 10 8-factor samples were considered in the statistical analyses. C) Principal coordinate analysis based on the taxonomic annotations of the reference viral MAGs. We indicate 8 factor samples not including the salinity treatment (124, 127, 128).

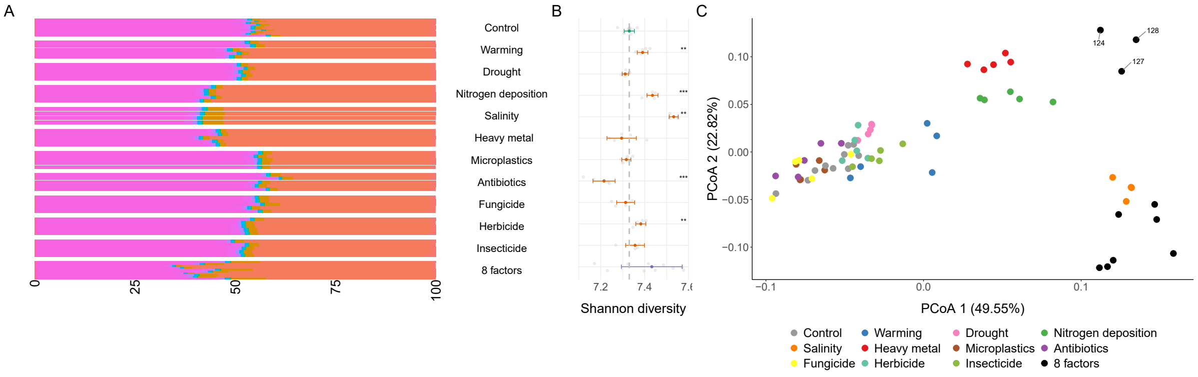

Supplementary Fig. 8. Prokaryotic community composition analysis taking Kraken2 [3] taxonomic profiles as a reference. A) Taxonomic profile collapsed to the phylum level. B) Shannon diversity index, per treatment. Thick points represent the median values, and bars indicate standard deviation intervals. Asterisks represent different significance levels obtained after a Two-sided Wilcoxon test comparing control samples with the samples to which GC treatments were applied; * indicate p <= 0.05, ** p <= 0.01, *** p <= 0.001 and **** p <= 0.0001. 10 Control samples, 5 samples for each individual GC treatment, and 10 8-factor samples were considered in the statistical analyses. C) Principal coordinate analysis based on the taxonomic annotations of the reference viral MAGs. We indicate 8 factor samples not including the salinity treatment (124, 127, 128).

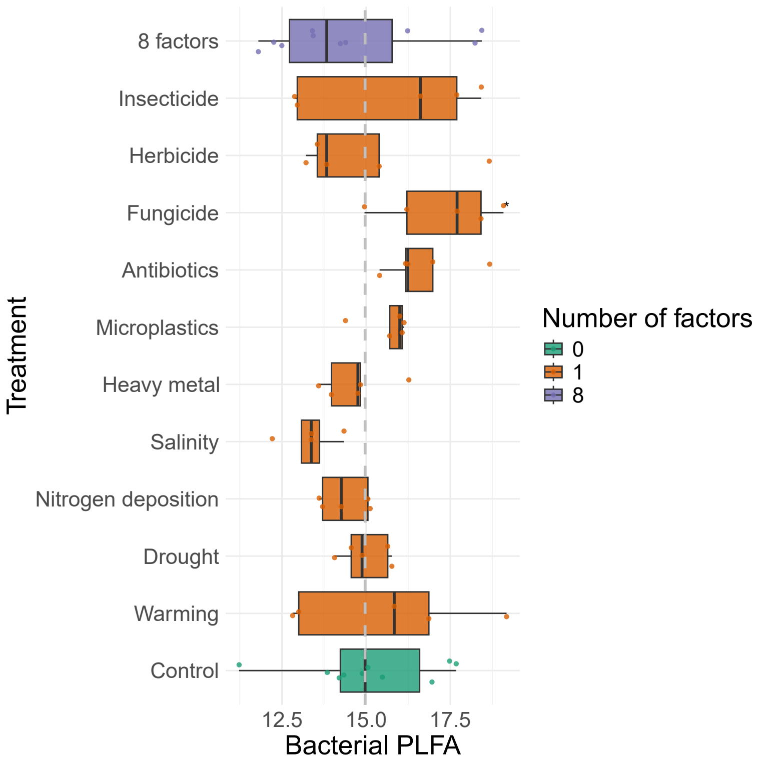

Supplementary Fig. 9. Bacterial PLFA values per GC treatment. Data are represented as boxplots in which the middle line is the median, the lower and upper hinges correspond to the first and third quartiles, the upper whisker extends from the hinge to the highest value no further than 1.5 × interquartile range (IQR) from the hinge and the lower whisker extends from the hinge to the lowest value no further than 1.5 × IQR of the hinge. Asterisks represent different significance levels obtained after a Two-sided Wilcoxon test comparing control samples with the samples to which GC treatments were applied; * indicate p <= 0.05, ** p <= 0.01, *** p <= 0.001 and **** p <= 0.0001. 10 Control samples, 5 samples for each individual GC treatment, and 10 8-factor samples were considered in the statistical analyses.

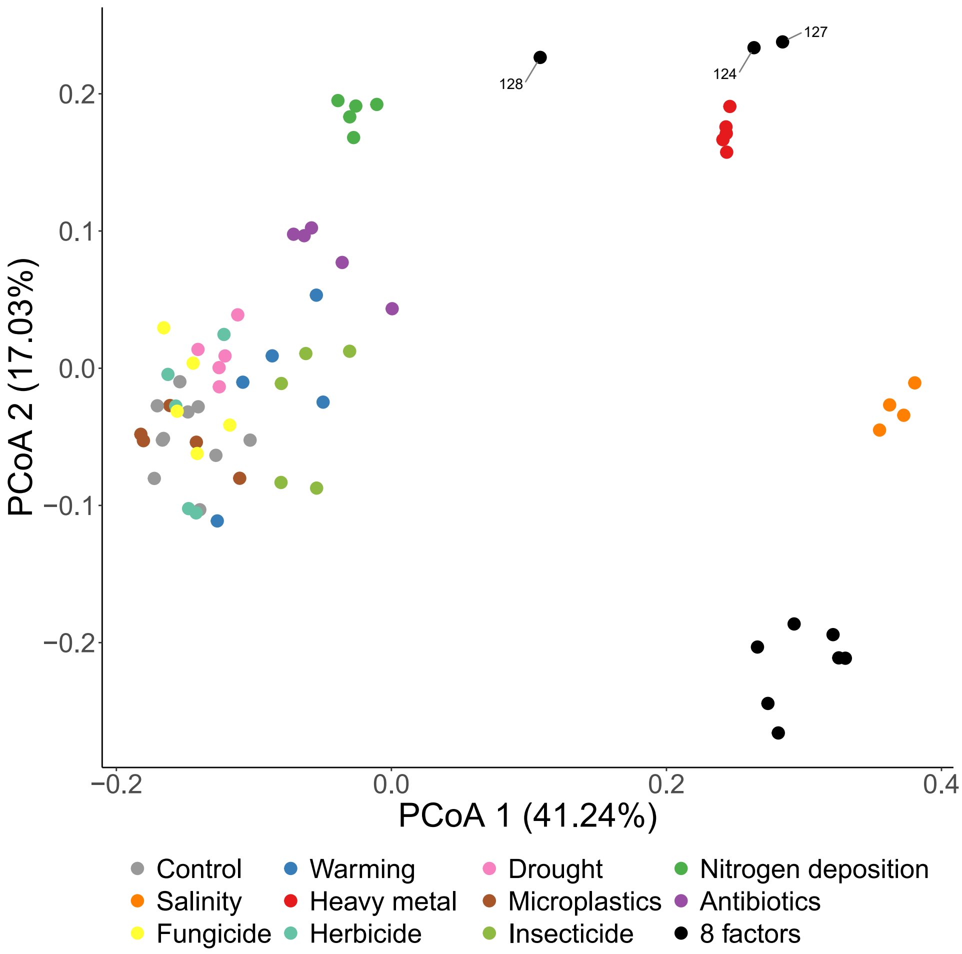

Supplementary Fig. 10. Prokaryotic beta diversity after correcting by bacterial PLFA per sample for calculating absolute abundances. We indicate 8 factor samples not including the salinity treatment (124, 127, 128).

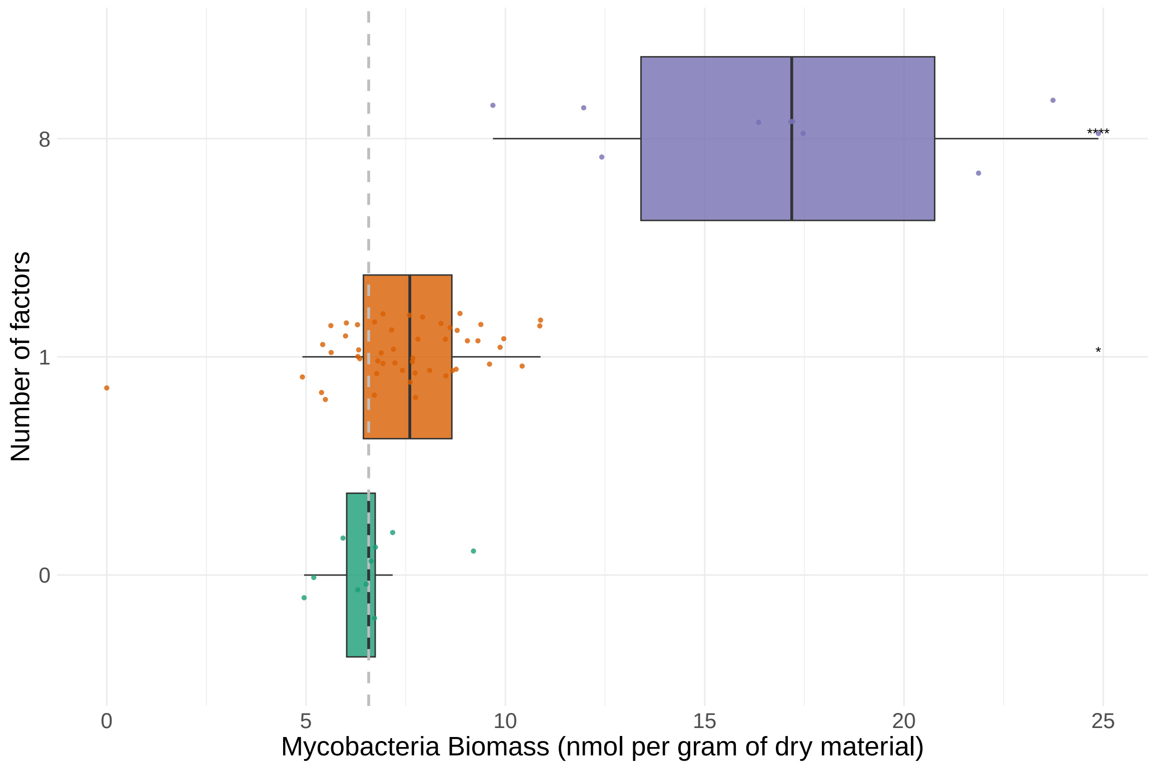

Supplementary Fig. 11. Biomass of unknown mycobacterial species (estimated by multiplying their relative abundances by bacterial PLFA values) per factor level. Data are represented as boxplots in which the middle line is the median, the lower and upper hinges correspond to the first and third quartiles, the upper whisker extends from the hinge to the highest value no further than 1.5 × interquartile range (IQR) from the hinge and the lower whisker extends from the hinge to the lowest value no further than 1.5 × IQR of the hinge. Asterisks represent different significance levels obtained after a Two-sided Wilcoxon test comparing control samples with the samples to which GC treatments were applied; * indicate $p <= 0.05$, ** $p <= 0.01$, *** $p <= 0.001$ and **** $p <= 0.0001$. 10 Control samples, 5 samples for each individual GC treatment, and 10 8-factor samples were considered in the statistical analyses.

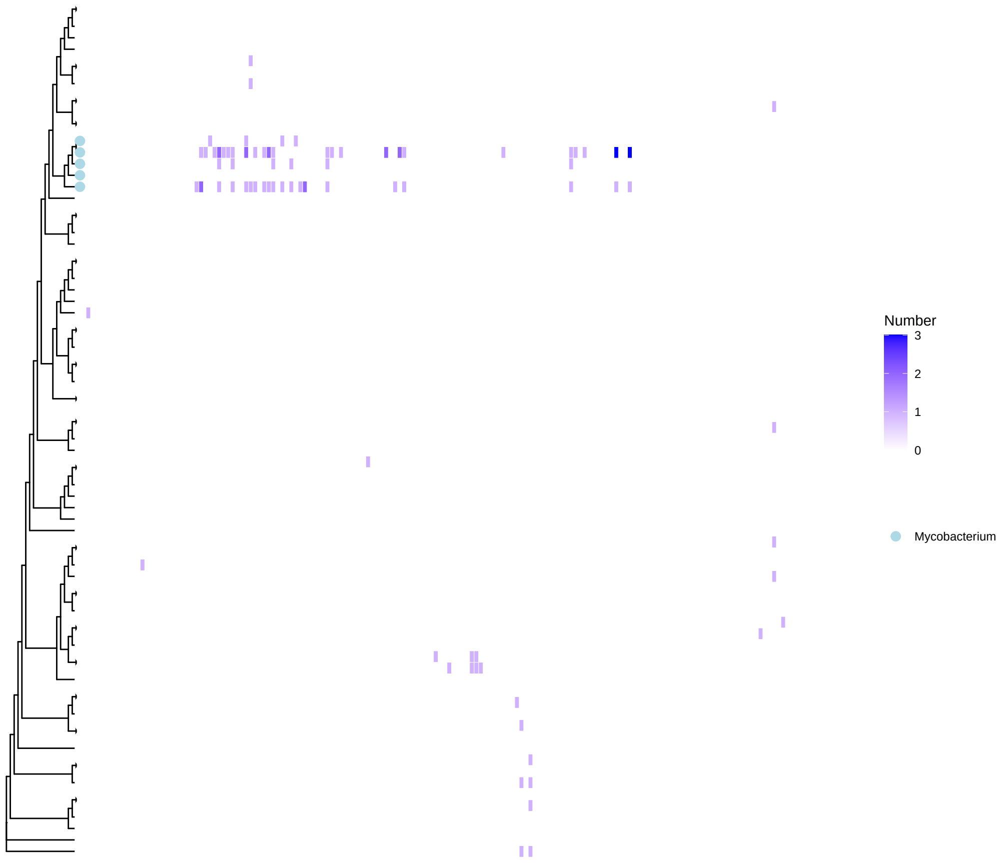

Supplementary Fig. 12. Virulence factor content (columns) per reference bin (rows). Blue tips point to the five *Mycobacterium* genomes.

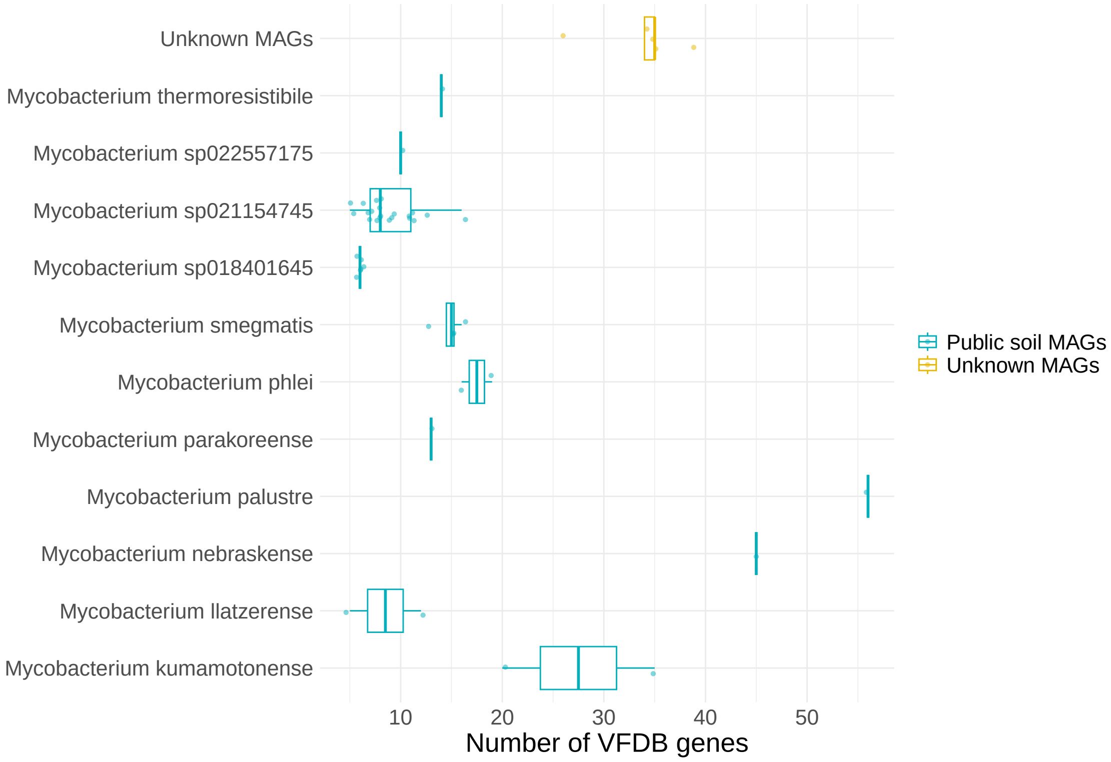

Supplementary Fig. 13. Comparison of virulence factor content within the VFDB [5] of the unknown *Mycobacterium* bins reconstructed here and *Mycobacterium* MAGs reconstructed from soil samples by Bin Ma et al. (2023)[6]. The density of virulence factors is similar to other NTM MAGs like Mycobacterium kumamotonense [7] and *Mycobacterium* phlei[8]. Data are represented as boxplots in which the middle line is the median, the lower and upper hinges correspond to the first and third quartiles, the upper whisker extends from the hinge to the highest value no further than 1.5 × interquartile range (IQR) from the hinge and the lower whisker extends from the hinge to the lowest value no further than 1.5 × IQR of the hinge.

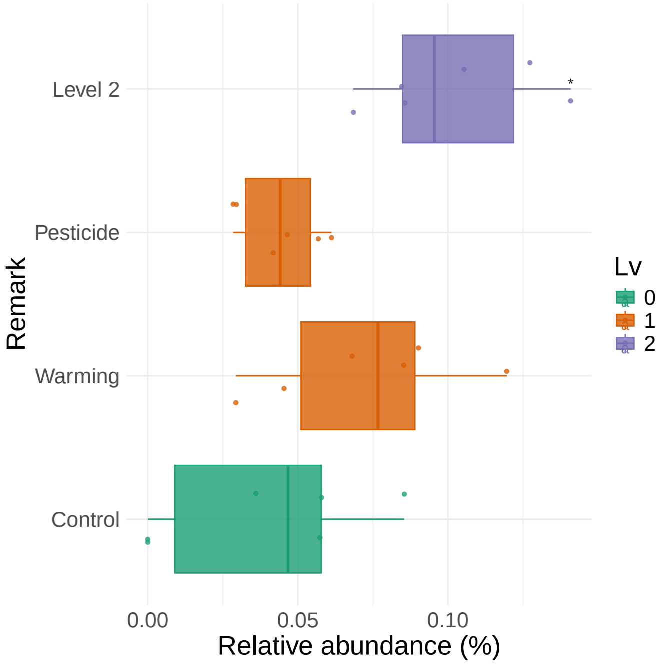

Supplementary Fig. 14. Relative abundance of the *Mycobacterium* ASVs located in the freshwater stressor experiment in Romero et al. (2020) [9]. Data are represented as boxplots in which the middle line is the median, the lower and upper hinges correspond to the first and third quartiles, the upper whisker extends from the hinge to the highest value no further than 1.5 × interquartile range (IQR) from the hinge and the lower whisker extends from the hinge to the lowest value no further than 1.5 × IQR of the hinge. Asterisks represent different significance levels obtained after a Two-sided Wilcoxon test with control samples; * indicate $p <= 0.05$, ** $p <= 0.01$, *** $p <= 0.001$ and **** $p <= 0.0001$. 6 samples for each category were considered in the statistical analyses.

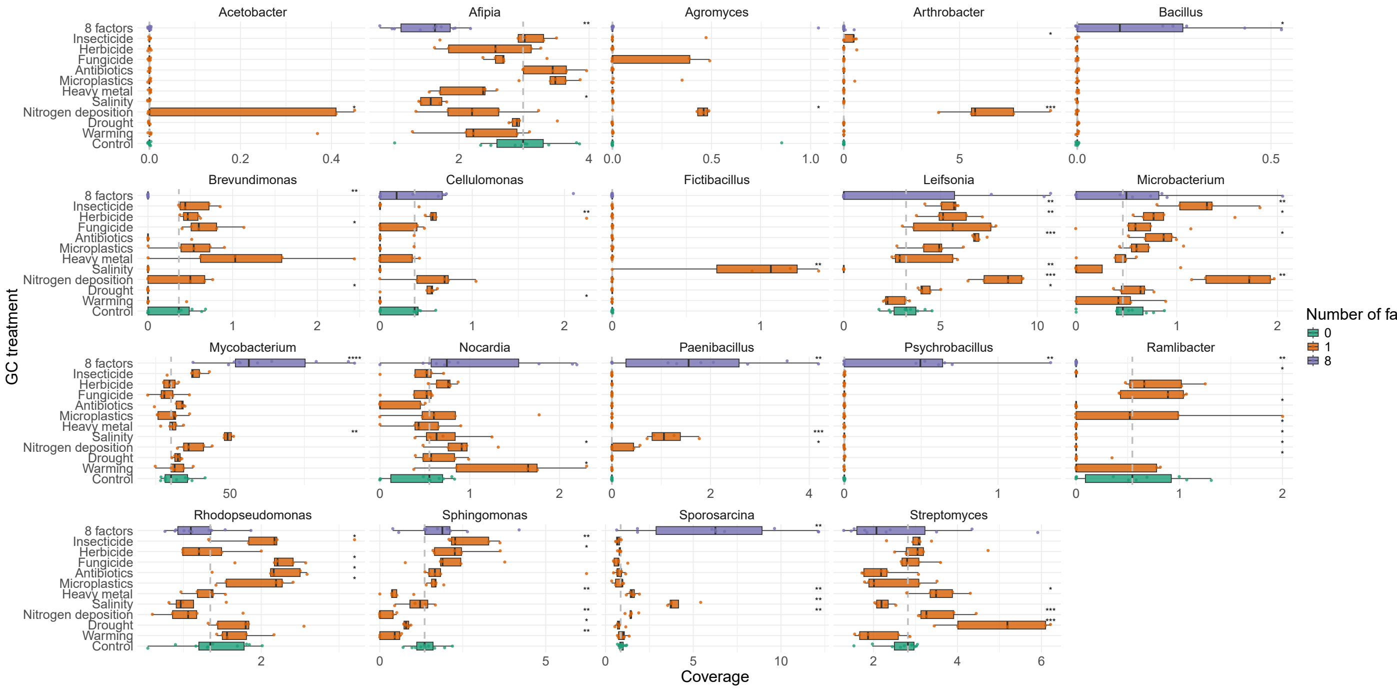

Supplementary Fig. 15. Relative abundance of genera (detected by SingleM[2]) within the MBPD database[10] that significantly shifted in abundance after one of the GC treatments (Two-sided Wilcoxon test, $p < 0.05$). Data are represented as boxplots in which the middle line is the median, the lower and upper hinges correspond to the first and third quartiles, the upper whisker extends from the hinge to the highest value no further than 1.5 × interquartile range (IQR) from the hinge and the lower whisker extends from the hinge to the lowest value no further than 1.5 × IQR of the hinge. Asterisks represent different significance levels obtained after a Two-sided Wilcoxon test comparing control samples with the samples to which GC treatments were applied; * indicate $p <= 0.05$, ** $p <= 0.01$, *** $p <= 0.001$ and **** $p <= 0.0001$. 10 Control samples, 5 samples for each individual GC treatment, and 10 8-factor samples were considered in the statistical analyses

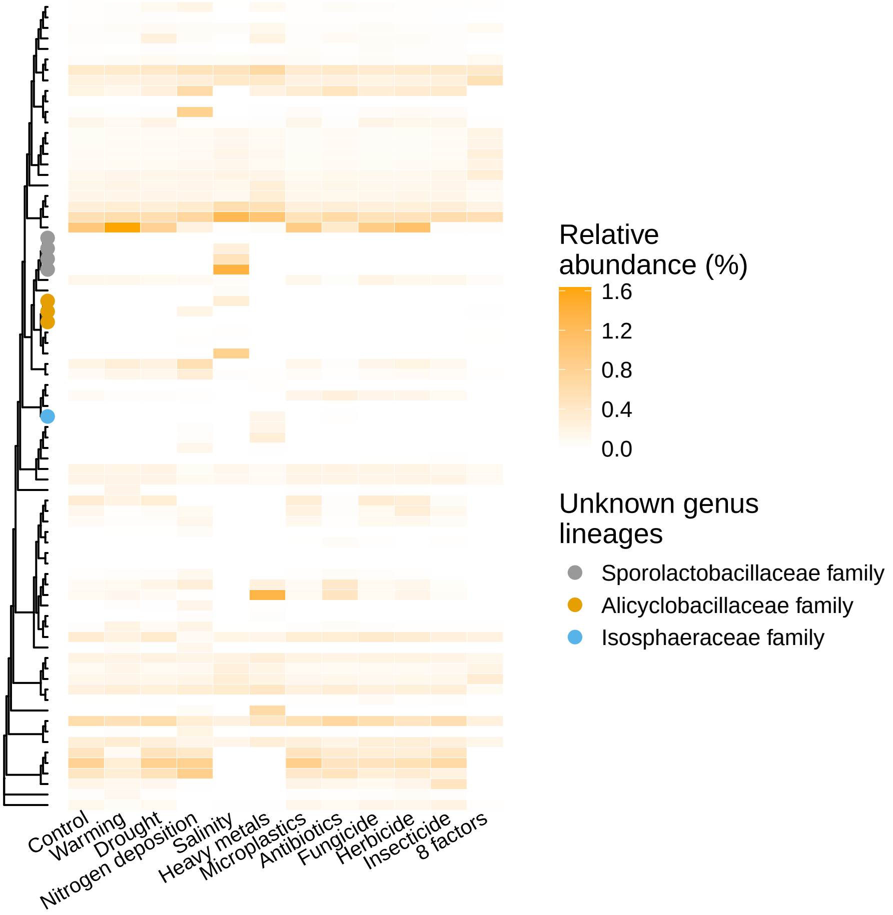

Supplementary Fig. 16. Mean relative abundance of MAGs in control samples and across GC treatments. We indicate genomes from unknown families non detected in control samples (median abundance = 0) increasing in abundance after some GC treatments.

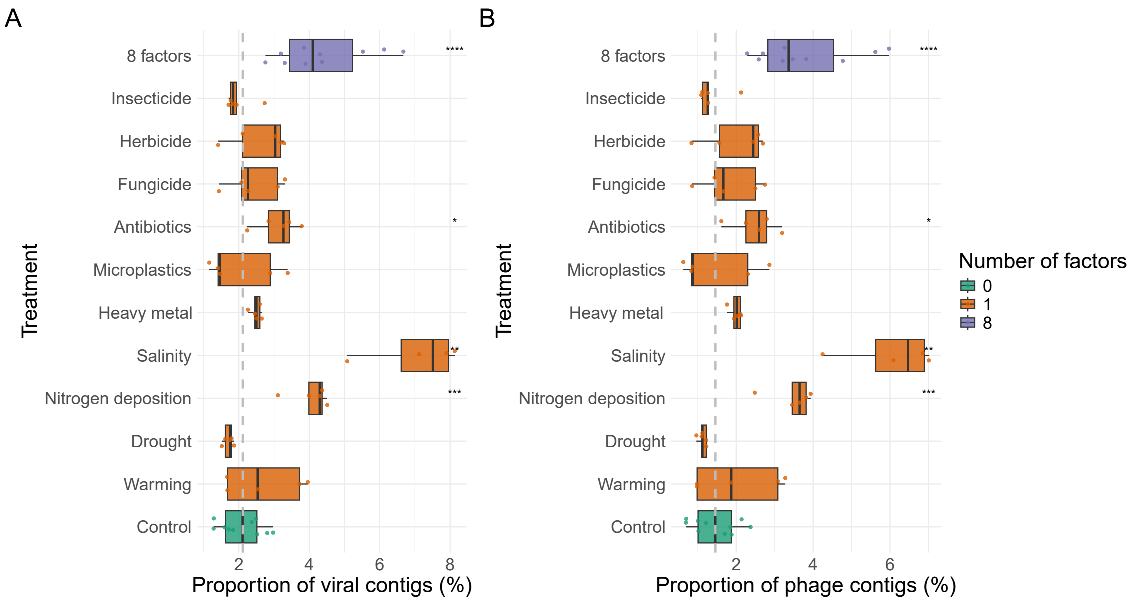

Supplementary Fig. 17. Proportion of A) viral contigs and B) phage contigs, per treatment. Data are represented as boxplots in which the middle line is the median, the lower and upper hinges correspond to the first and third quartiles, the upper whisker extends from the hinge to the highest value no further than 1.5 × interquartile range (IQR) from the hinge and the lower whisker extends from the hinge to the lowest value no further than 1.5 × IQR of the hinge. Asterisks represent different significance levels obtained after a Two-sided Wilcoxon test comparing control samples with the samples to which GC treatments were applied; * indicate $p <= 0.05$, ** $p <= 0.01$, *** $p <= 0.001$ and **** $p <= 0.0001$. 10 Control samples, 5 samples for each individual GC treatment, and 10 8-factor samples were considered in the statistical analyses.

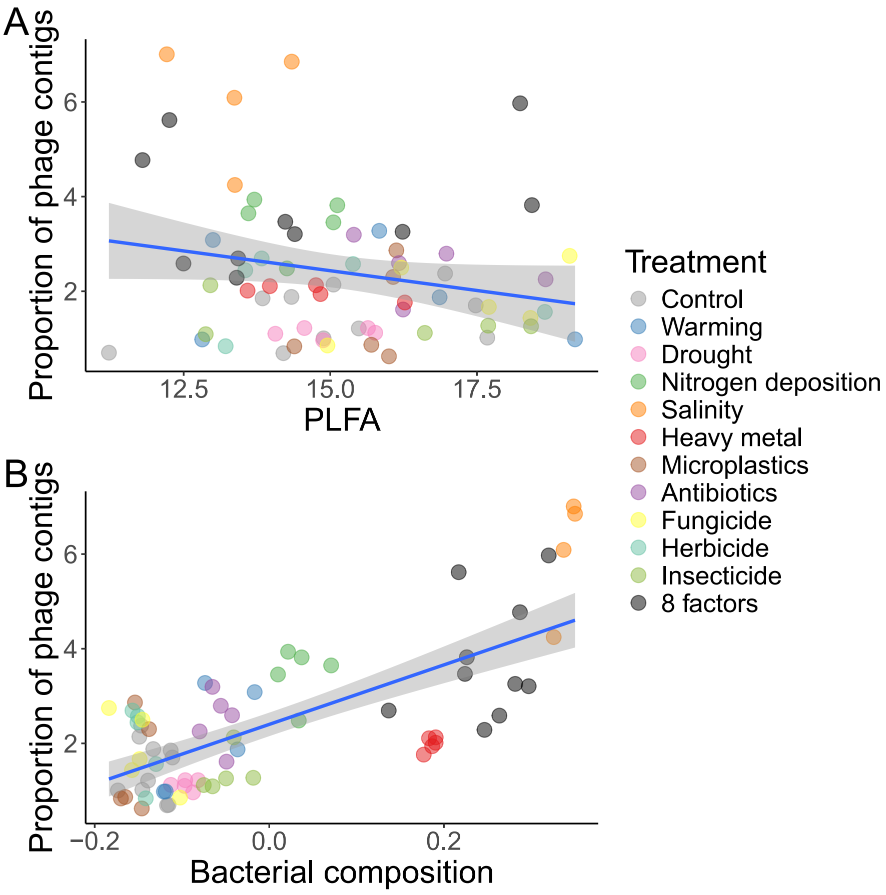

Supplementary Fig. 18. Phage correlations with bacterial biomass and composition. A) Correlation between bacterial PLFA and the proportion of phages. B) Correlation between bacterial composition and the proportion of phages. Blue lines represent linear regression lines, and shaded areas indicate 95% confidence intervals.

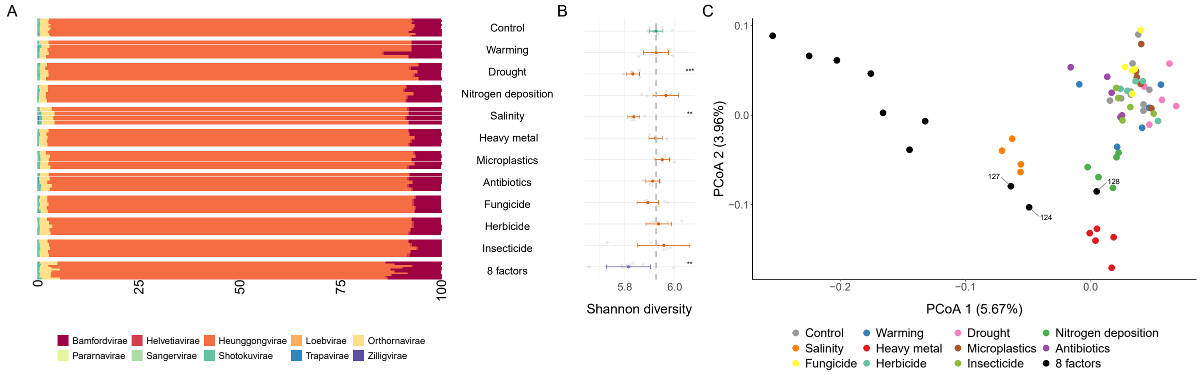

Supplementary Fig. 19. Viral community composition and diversity assessed by Kraken2 [3]. A) Taxonomic profile of the representative viral MAGs reconstructed from the 70 samples included in this analysis (10 controls, 5 for 10 different GC factors, 10 random combinations of 8 factors) collapsed to the kingdom level. B) Shannon diversity index, per treatment. Thick points represent the median values, and bars indicate standard deviation intervals. Asterisks represent different significance levels obtained after a Two-sided Wilcoxon test comparing control samples with the samples to which GC treatments were applied; * indicate p <= 0.05, ** p <= 0.01, *** p <= 0.001 and **** p <= 0.0001. 10 Control samples, 5 samples for each individual GC treatment, and 10 8-factor samples were considered in the statistical analyses. C) Principal coordinate analysis based on the taxonomic annotations of the reference viral MAGs. We indicate 8 factor samples not including the salinity treatment (124, 127, 128).

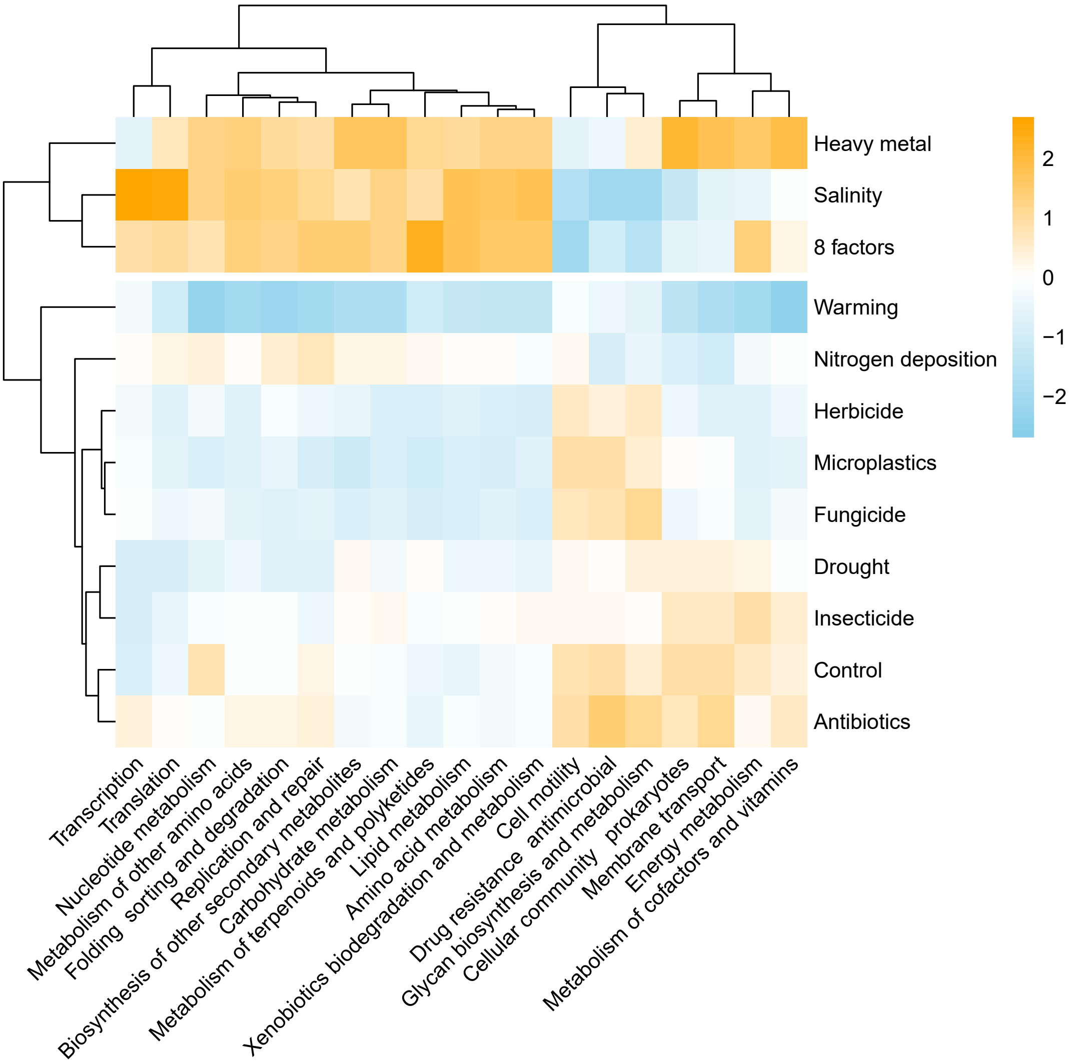

Supplementary Fig. 20. Heatmap indicating the copy number per cell, normalized by column, of general KEGG [11] pathways.

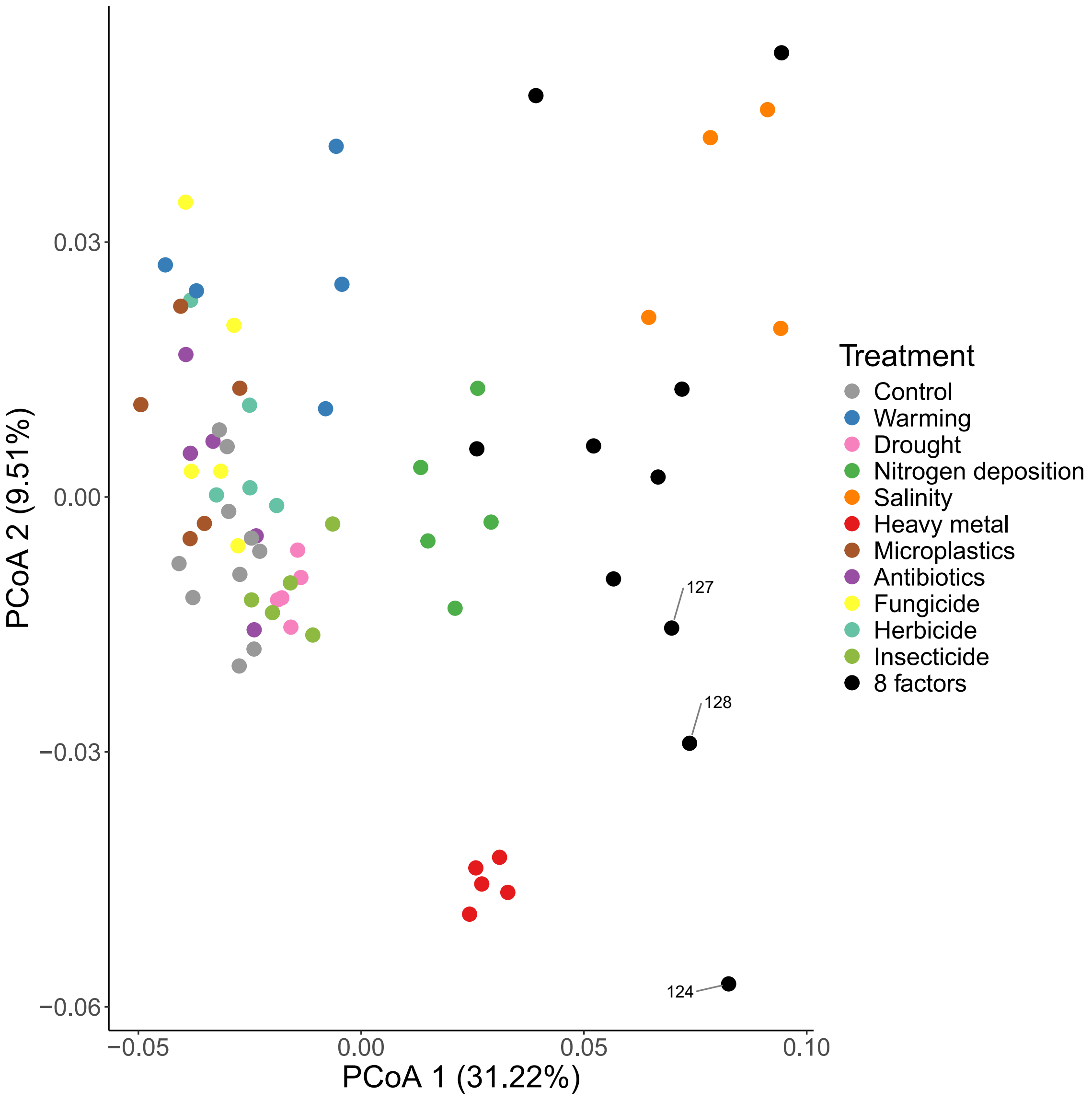

Supplementary Fig. 21. PCoA built on the frequency of KEGG[11] Orthologues (KOs). We indicate samples 124, 127 and 128, missing the salinity treatment

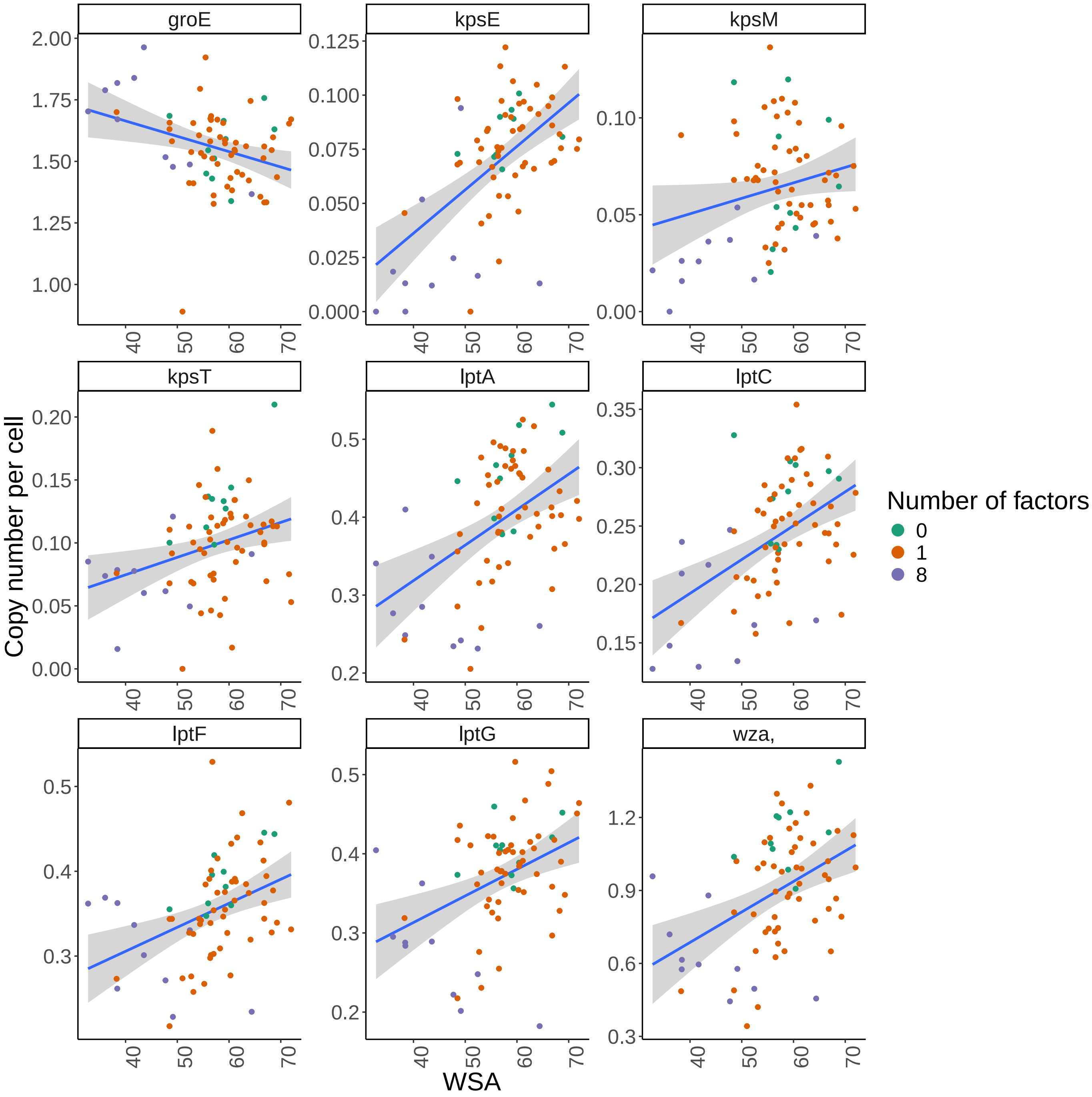

Supplementary Fig. 22. Correlation of genes previously associated with Water Stable Aggregates (WSA), with WSA measures. Blue lines represent linear regression lines, and shaded areas indicate 95% confidence intervals

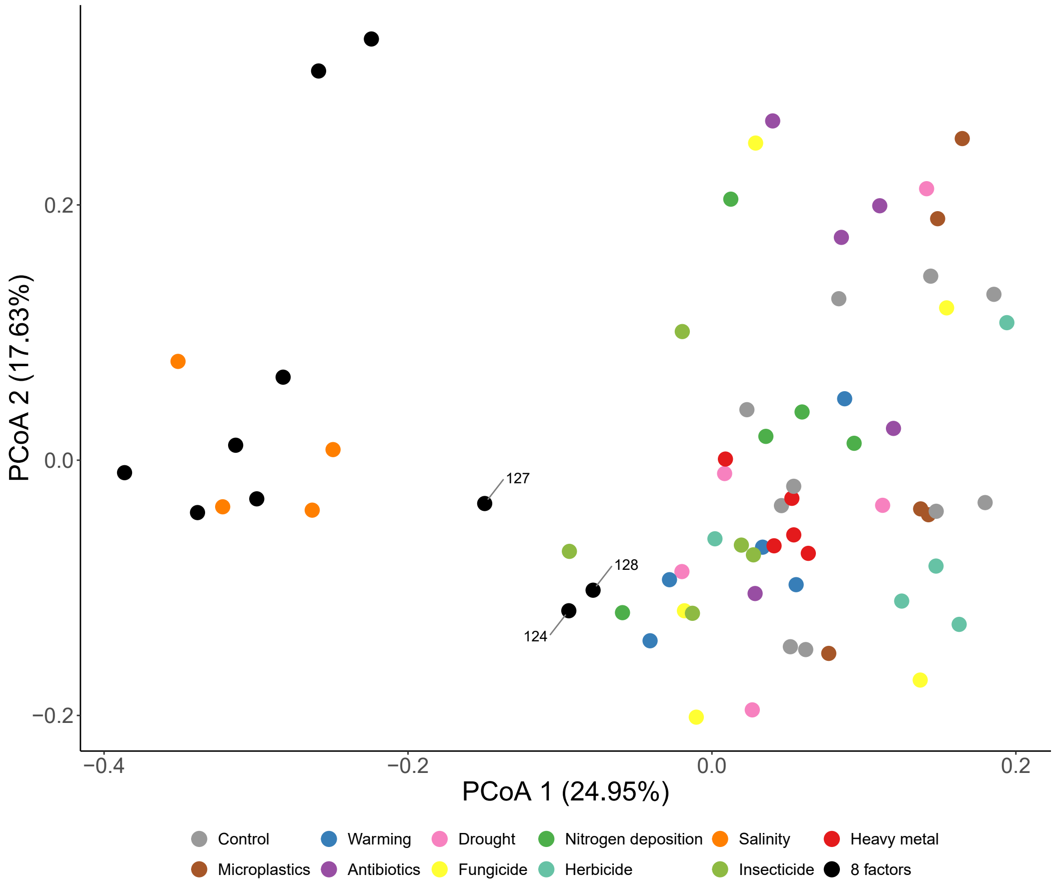

Supplementary Fig. 23. PCoA built on the frequency of CARD [12] genes. We indicate samples 124, 127 and 128, missing the salinity treatment

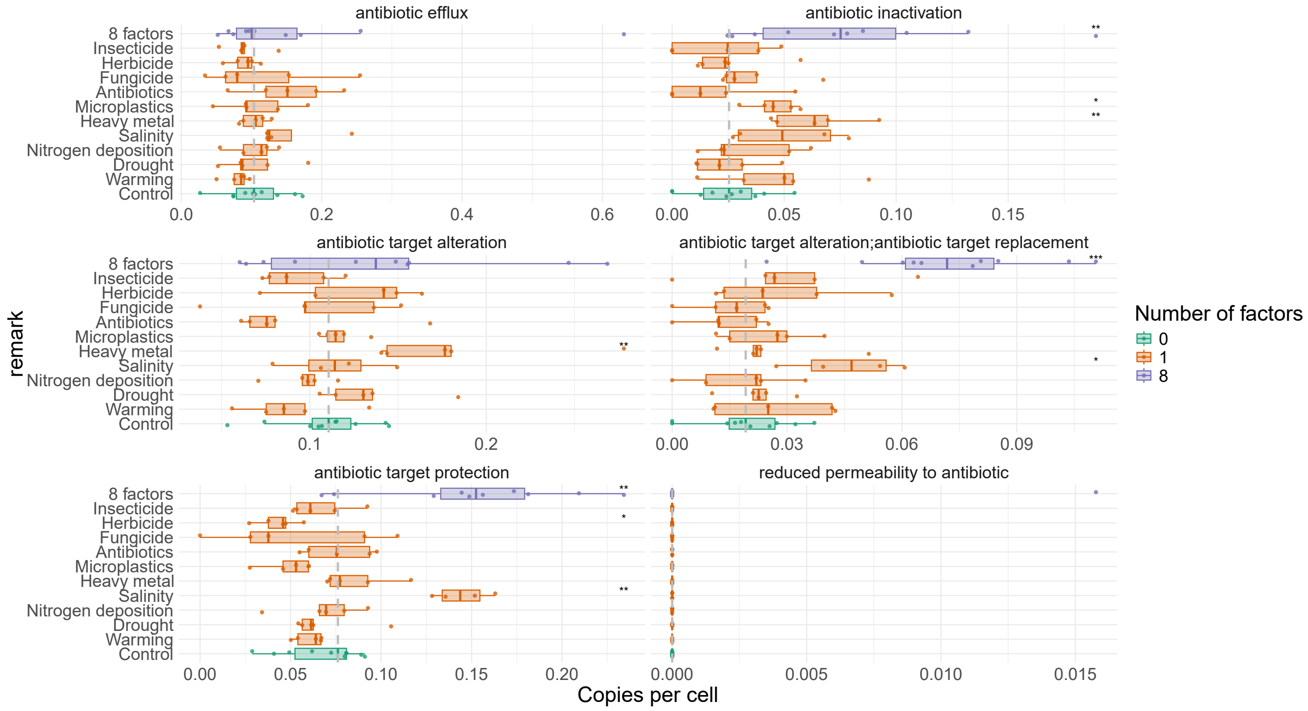

Supplementary Fig. 24. Copy number per cell of all categories included in the CARD database [12]. Data are represented as boxplots in which the middle line is the median, the lower and upper hinges correspond to the first and third quartiles, the upper whisker extends from the hinge to the highest value no further than 1.5 × interquartile range (IQR) from the hinge and the lower whisker extends from the hinge to the lowest value no further than 1.5 × IQR of the hinge. Asterisks represent different significance levels obtained after a Two-sided Wilcoxon test comparing control samples with the samples to which GC treatments were applied; * indicate $p <= 0.05$, ** $p <= 0.01$, *** $p <= 0.001$ and **** $p <= 0.0001$. 10 Control samples, 5 samples for each individual GC treatment, and 10 8-factor samples were considered in the statistical analyses. We do not report the results on the "antibiotic target alteration" category in the main figure because these may be gene variants with high degree of similarity which we cannot distinguish with the identity thresholds applied. We also did not include antibiotic efflux in the main figure because no significant changes were observed.

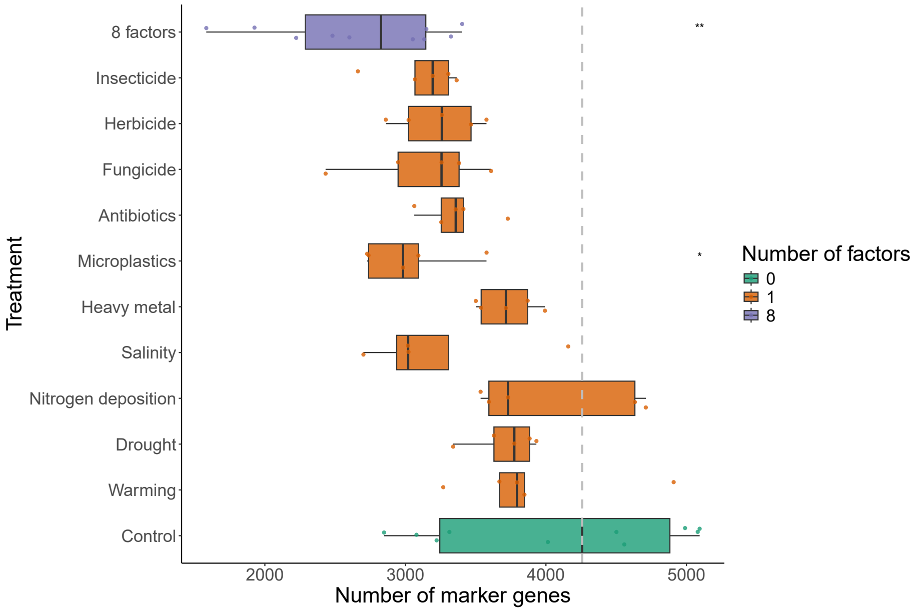

Supplementary Fig. 25. Number of assembled marker genes per treatment. Data are represented as boxplots in which the middle line is the median, the lower and upper hinges correspond to the first and third quartiles, the upper whisker extends from the hinge to the highest value no further than 1.5 × interquartile range (IQR) from the hinge and the lower whisker extends from the hinge to the lowest value no further than 1.5 × IQR of the hinge. Asterisks represent different significance levels obtained after a Two-sided Wilcoxon test comparing control samples with the samples to which GC treatments were applied; * indicate $p <= 0.05$, ** $p <= 0.01$, *** $p <= 0.001$ and **** $p <= 0.0001$. 10 Control samples, 5 samples for each individual GC treatment, and 10 8-factor samples were considered in the statistical analyses.

Supplementary Table 1: Comparison of High-Quality (HQ) and Medium-Quality (MQ) bins across different software tools.

Software	Number of HQ bins	Number of MQ bins
Semibin2	89	742
Metabat2	10	99
Maxbin2	52	357
MAGScoT	98	562
MAGScoT (relaxed options)	101	625

Supplementary Table 2: P-values for the two sided Wilcoxon test comparing the abundance of *Bradyrhizobium* in control samples and in samples applied different GC treatments

Treatment	p-value
Antibiotics	0.00799
Heavy metal	0.00133
Drought	0.513
Microplastics	0.0280
Nitrogen deposition	0.0193
Salinity	0.00200
Warming	0.206
Fungicide	0.206
Herbicide	0.000666
Insecticide	0.0280
8 factors	0.0000108

Supplementary Table 3: P-values for the two sided Wilcoxon test comparing Shannon diversity in control samples and in samples applied different GC treatments

Treatment	p-value
8 factors	0.0000108
Insecticide	0.0400
Herbicide	0.0127
Fungicide	0.00133
Antibiotics	0.679
Microplastics	0.206
Heavy metal	0.000666
Salinity	0.00200
Nitrogen deposition	0.00466
Drought	0.0127
Warming	0.440

Supplementary Table 4: P-values for the two sided Wilcoxon test comparing the genome sizes (corrected by completness) of genomic bins reconstructed from control samples and from samples applied different GC treatments

Treatment	p-value
8 factors	0.00404
Insecticide	0.846
Herbicide	0.741
Fungicide	0.390
Antibiotics	0.609
Microplastics	0.444
Heavy metal	0.00000852
Salinity	0.609
Nitrogen deposition	0.983
Drought	0.610
Warming	0.528

Supplementary Table 5: P-values for the two sided Wilcoxon test comparing the abundance of *Mycobacterium* in control samples and in samples applied different GC treatments

Treatment	p-value
Antibiotics	0.440
Heavy metal	0.0127
Drought	0.0400
Microplastics	0.440
Nitrogen deposition	0.0127
Salinity	0.00200
Warming	0.310
Fungicide	0.859
Herbicide	1
Insecticide	0.0753
8 factors	0.0000108

Supplementary Table 6: P-values for the two-sided Wilcoxon test comparing the abundance of *Mycobacterium* species in control samples and in samples under different GC treatments. Only species with different abundances (two-sided Wilcoxon test $p < 0.05$) in control and 8-factor samples are shown.

Treatment	Species	p-value
Warming	Mycobacterium colombiense [ref_mOTU_v31_01274]	0.340
Drought	Mycobacterium colombiense [ref_mOTU_v31_01274]	0.284
Nitrogen deposition	Mycobacterium colombiense [ref_mOTU_v31_01274]	0.340
Salinity	Mycobacterium colombiense [ref_mOTU_v31_01274]	0.128
Heavy metal	Mycobacterium colombiense [ref_mOTU_v31_01274]	0.210
Microplastics	Mycobacterium colombiense [ref_mOTU_v31_01274]	0.448
Antibiotics	Mycobacterium colombiense [ref_mOTU_v31_01274]	0.850
Fungicide	Mycobacterium colombiense [ref_mOTU_v31_01274]	0.301
Herbicide	Mycobacterium colombiense [ref_mOTU_v31_01274]	0.611
Insecticide	Mycobacterium colombiense [ref_mOTU_v31_01274]	0.850
8 factors	Mycobacterium colombiense [ref_mOTU_v31_01274]	0.0103
Warming	Mycobacterium colombiense [ref_mOTU_v31_01275]	0.742
Drought	Mycobacterium colombiense [ref_mOTU_v31_01275]	0.384
Nitrogen deposition	Mycobacterium colombiense [ref_mOTU_v31_01275]	0.312
Salinity	Mycobacterium colombiense [ref_mOTU_v31_01275]	0.705
Heavy metal	Mycobacterium colombiense [ref_mOTU_v31_01275]	0.643

Treatment	Species	p-value
Microplastics	Mycobacterium colombiense [ref_mOTU_v31_01275]	1
Antibiotics	Mycobacterium colombiense [ref_mOTU_v31_01275]	0.397
Fungicide	Mycobacterium colombiense [ref_mOTU_v31_01275]	0.650
Herbicide	Mycobacterium colombiense [ref_mOTU_v31_01275]	0.745
Insecticide	Mycobacterium colombiense [ref_mOTU_v31_01275]	0.397
8 factors	Mycobacterium colombiense [ref_mOTU_v31_01275]	0.0334
Warming	Mycobacterium mantenii [ref_mOTU_v31_01272]	0.896
Drought	Mycobacterium mantenii [ref_mOTU_v31_01272]	0.840
Nitrogen deposition	Mycobacterium mantenii [ref_mOTU_v31_01272]	0.443
Salinity	Mycobacterium mantenii [ref_mOTU_v31_01272]	0.410
Heavy metal	Mycobacterium mantenii [ref_mOTU_v31_01272]	0.497
Microplastics	Mycobacterium mantenii [ref_mOTU_v31_01272]	0.366
Antibiotics	Mycobacterium mantenii [ref_mOTU_v31_01272]	0.443
Fungicide	Mycobacterium mantenii [ref_mOTU_v31_01272]	0.365
Herbicide	Mycobacterium mantenii [ref_mOTU_v31_01272]	0.896
Insecticide	Mycobacterium mantenii [ref_mOTU_v31_01272]	0.225
8 factors	Mycobacterium mantenii [ref_mOTU_v31_01272]	0.0232
Warming	Mycobacterium sp. 1245111.1 [ref_mOTU_v31_10092]	0.749
Drought	Mycobacterium sp. 1245111.1 [ref_mOTU_v31_10092]	0.0743
Nitrogen deposition	Mycobacterium sp. 1245111.1 [ref_mOTU_v31_10092]	0.0235
Salinity	Mycobacterium sp. 1245111.1 [ref_mOTU_v31_10092]	0.0330
Heavy metal	Mycobacterium sp. 1245111.1 [ref_mOTU_v31_10092]	0.523
Microplastics	Mycobacterium sp. 1245111.1 [ref_mOTU_v31_10092]	0.432
Antibiotics	Mycobacterium sp. 1245111.1 [ref_mOTU_v31_10092]	0.0467
Fungicide	Mycobacterium sp. 1245111.1 [ref_mOTU_v31_10092]	0.0360
Herbicide	Mycobacterium sp. 1245111.1 [ref_mOTU_v31_10092]	0.0956
Insecticide	Mycobacterium sp. 1245111.1 [ref_mOTU_v31_10092]	0.0564
8 factors	Mycobacterium sp. 1245111.1 [ref_mOTU_v31_10092]	0.0101
Warming	Mycobacterium sp. E2479 [ref_mOTU_v31_01279]	0.197
Drought	Mycobacterium sp. E2479 [ref_mOTU_v31_01279]	0.485
Nitrogen deposition	Mycobacterium sp. E2479 [ref_mOTU_v31_01279]	0.241
Salinity	Mycobacterium sp. E2479 [ref_mOTU_v31_01279]	0.104
Heavy metal	Mycobacterium sp. E2479 [ref_mOTU_v31_01279]	0.624
Microplastics	Mycobacterium sp. E2479 [ref_mOTU_v31_01279]	0.621
Antibiotics	Mycobacterium sp. E2479 [ref_mOTU_v31_01279]	0.0610
Fungicide	Mycobacterium sp. E2479 [ref_mOTU_v31_01279]	0.270
Herbicide	Mycobacterium sp. E2479 [ref_mOTU_v31_01279]	0.547
Insecticide	Mycobacterium sp. E2479 [ref_mOTU_v31_01279]	0.0870
8 factors	Mycobacterium sp. E2479 [ref_mOTU_v31_01279]	0.0486
Warming	Mycobacterium sp. [ref_mOTU_v31_02613]	0.580
Drought	Mycobacterium sp. [ref_mOTU_v31_02613]	0.237
Nitrogen deposition	Mycobacterium sp. [ref_mOTU_v31_02613]	0.0589
Salinity	Mycobacterium sp. [ref_mOTU_v31_02613]	1
Heavy metal	Mycobacterium sp. [ref_mOTU_v31_02613]	1
Microplastics	Mycobacterium sp. [ref_mOTU_v31_02613]	0.352
Antibiotics	Mycobacterium sp. [ref_mOTU_v31_02613]	0.580
Fungicide	Mycobacterium sp. [ref_mOTU_v31_02613]	1
Herbicide	Mycobacterium sp. [ref_mOTU_v31_02613]	0.580
Insecticide	Mycobacterium sp. [ref_mOTU_v31_02613]	1
8 factors	Mycobacterium sp. [ref_mOTU_v31_02613]	0.0226
Warming	Mycobacterium sp. [ref_mOTU_v31_04172]	0.678
Drought	Mycobacterium sp. [ref_mOTU_v31_04172]	0.678
Nitrogen deposition	Mycobacterium sp. [ref_mOTU_v31_04172]	0.678
Salinity	Mycobacterium sp. [ref_mOTU_v31_04172]	0.137
Heavy metal	Mycobacterium sp. [ref_mOTU_v31_04172]	0.572
Microplastics	Mycobacterium sp. [ref_mOTU_v31_04172]	0.572

Treatment	Species	p-value
Antibiotics	Mycobacterium sp. [ref_mOTU_v31_04172]	0.572
Fungicide	Mycobacterium sp. [ref_mOTU_v31_04172]	0.678
Herbicide	Mycobacterium sp. [ref_mOTU_v31_04172]	0.572
Insecticide	Mycobacterium sp. [ref_mOTU_v31_04172]	0.572
8 factors	Mycobacterium sp. [ref_mOTU_v31_04172]	0.0222

Supplementary Table 7: P-values for the two sided Wilcoxon test comparing the abundance of viral contigs in control samples and in samples applied different GC treatments

Treatment	p-value
Warming	0.310
Drought	0.440
Nitrogen deposition	0.000666
Salinity	0.00200
Heavy metal	0.310
Microplastics	0.859
Antibiotics	0.0193
Fungicide	0.440
Herbicide	0.165
Insecticide	0.953
8 factors	0.0000433

Supplementary Table 8: P-values for the two sided Wilcoxon test comparing the viral Shannon diversity in control samples and in samples applied different GC treatments

Treatment	p-value
2 Antibiotics	0.00266
3 Heavy metal	0.513
4 Drought	0.310
5 Microplastics	0.129
6 Nitrogen deposition	0.00466
7 Salinity	0.00200
8 Warming	0.254
9 Fungicide	0.0280
10 Herbicide	0.00466
11 Insecticide	0.129
12 8 factors	0.796

Supplementary Table 9: P-values for the two sided Wilcoxon test comparing the abundance of antibiotic target protection and antibiotic inactivation genes in control samples and in samples applied different GC treatments

**Treatment**	**ARG category**	**p-value**
Warming	antibiotic target protection	0.440
Drought	antibiotic target protection	0.859
Nitrogen deposition	antibiotic target protection	0.953
Salinity	antibiotic target protection	0.00200
Heavy metal	antibiotic target protection	0.371
Microplastics	antibiotic target protection	0.0992
Antibiotics	antibiotic target protection	0.513
Fungicide	antibiotic target protection	0.594
Herbicide	antibiotic target protection	0.0400
Insecticide	antibiotic target protection	0.953
8 factors	antibiotic target protection	0.00209
Warming	antibiotic inactivation	0.159
Drought	antibiotic inactivation	0.951
Nitrogen deposition	antibiotic inactivation	0.668
Salinity	antibiotic inactivation	0.103
Heavy metal	antibiotic inactivation	0.00581
Microplastics	antibiotic inactivation	0.0319
Antibiotics	antibiotic inactivation	0.422
Fungicide	antibiotic inactivation	0.358
Herbicide	antibiotic inactivation	0.951
Insecticide	antibiotic inactivation	0.951
8 factors	antibiotic inactivation	0.00578

Supplementary Table 10: P-values for the two sided Wilcoxon test comparing the abundance of BJP-1 and RbpA genes in control samples and in samples applied different GC treatments

**Treatment**	**ARG gene**	**p-value**
Warming	RbpA	0.440
Drought	RbpA	0.254
Nitrogen deposition	RbpA	0.0193
Salinity	RbpA	0.00200
Heavy metal	RbpA	0.859
Microplastics	RbpA	0.0992
Antibiotics	RbpA	0.859
Fungicide	RbpA	0.679
Herbicide	RbpA	0.679
Insecticide	RbpA	0.0753
8 factors	RbpA	0.0000433
Warming	BJP-1	0.0935
Drought	BJP-1	0.307
Nitrogen deposition	BJP-1	0.0226
Salinity	BJP-1	0.184
Heavy metal	BJP-1	0.307
Microplastics	BJP-1	0.352
Antibiotics	BJP-1	0.307
Fungicide	BJP-1	0.307
Herbicide	BJP-1	0.930
Insecticide	BJP-1	0.307
8 factors	BJP-1	0.00915

References

1. Nayfach, S. & Pollard, K. S. Average genome size estimation improves comparative metagenomics and sheds light on the functional ecology of the human microbiome. Genome Biol. 16, 51 (2015).
2. Woodcroft, B. J. et al. SingleM and Sandpiper: Robust microbial taxonomic profiles from metagenomic data. bioRxiv 2024.01.30.578060 (2024) doi:10.1101/2024.01.30.578060.
3. Wood, D. E., Lu, J. & Langmead, B. Improved metagenomic analysis with Kraken 2. Genome Biol. 20, 257 (2019).
4. Ruscheweyh, H.-J. et al. Cultivation-independent genomes greatly expand taxonomic-profiling capabilities of mOTUs across various environments. Microbiome 10, 212 (2022).
5. Chen, L. et al. VFDB: a reference database for bacterial virulence factors. Nucleic Acids Res. 33, D325–8 (2005).
6. Ma, B. et al. A genomic catalogue of soil microbiomes boosts mining of biodiversity and genetic resources. Nat. Commun. 14, 7318 (2023).
7. Kontos, F., Mavromanolakis, D. N., Zande, M. C. & Gitti, Z. G. Isolation of Mycobacterium kumamotonense from a patient with pulmonary infection and latent tuberculosis. Indian J. Med. Microbiol. 34, 241–244 (2016).
8. Sánchez Ramos, D., Pinto Plá, C., De Gracia León, A. & Colomina Rodríguez, J. Rare infectious complication after intramuscular self-injections. Rev. Esp. Quimioter. 34, 393–395 (2021).
9. Romero, F., Acuña, V. & Sabater, S. Multiple Stressors Determine Community Structure and Estimated Function of River Biofilm Bacteria. Appl. Environ. Microbiol. 86, (2020).
10. Yang, X. et al. MBPD: A multiple bacterial pathogen detection pipeline for One Health practices. Imeta 2, e82 (2023).
11. Kanehisa, M. & Goto, S. KEGG: kyoto encyclopedia of genes and genomes. Nucleic Acids Res. 28, 27–30 (2000).
12. Alcock, B. P. et al. CARD 2020: antibiotic resistome surveillance with the comprehensive antibiotic resistance database. Nucleic Acids Res. 48, D517–D525 (2020).
