# Supplementary material for: Soil microbial responses to multiple global change factors as assessed by metagenomics
Source: Nat Commun. 2025 May 31;16:5058. doi: 10.1038/s41467-025-60390-4 (PMC12125317; doi:10.1038/s41467-025-60390-4)
Supplement: Supplementary file 2 — Description of Additional Supplementary Files [file 41467_2025_60390_MOESM2_ESM.pdf]

Description of Additional Supplementary Files

Supplementary Data 1. Genes enriched in conditionally rare bins detected after the salinity treatment with q-value < 1e-20.

Supplementary Data 2. Genes enriched in conditionally rare bins detected after the heavy metal treatment with q-value < 1e-2.
